# Factors in Early Feeding Practices That May Influence Growth and the Challenges That Arise in Growth Outcomes Research

**DOI:** 10.3390/nu12071939

**Published:** 2020-06-30

**Authors:** Veronica Fabrizio, Veronika Shabanova, Sarah N. Taylor

**Affiliations:** 1 Connecticut Children’s, Division of Neonatology, Hartford, CT 06106, USA; 2 Department of Pediatrics, University of Connecticut School of Medicine, Farmington, CT 06032, USA; 3 Department of Pediatrics, Yale School of Medicine, New Haven, CT 06443, USA; veronika.shabanova@yale.edu (V.S.); sarah.n.taylor@yale.edu (S.N.T.)

**Keywords:** preterm, growth, *z*-score, nutrition

## Abstract

Growth in preterm infants is associated with improved outcomes during both the initial hospitalization and long-term. A single center investigation evaluated preterm infant in-hospital growth trajectory and how it related to early nutritional practices as a way to identify areas for quality improvement. Infants born <34 weeks’ gestational age and with discharge at or prior to 40 weeks’ gestational age were included and were divided into quartiles based on their change in weight *z*-score through hospitalization. Early nutritional factors were compared between the lowest and highest growth quartiles. Although the highest growth quartile experienced less days of parenteral nutrition and higher enteral caloric density in the first week, as the data was analyzed, potential statistical pitfalls became apparent. Therefore, the aim of this study was to investigate selection bias and cohort effect related to both the inclusion criteria for this cohort as well as the inherent challenges in comparison of preterm infant growth trajectories by standard fetal growth charts.

## 1. Introduction

Measuring postnatal growth is a critical component of preterm infant hospital care, and growth trajectory before term equivalent age is linked to neurodevelopmental outcomes [1,2,3,4,5]. Yet, postnatal growth failure and lack of appropriate methods of measuring growth remain a significant challenge in preterm infant care. Despite many advancements in neonatal care, postnatal growth failure remains a major comorbidity in preterm infants, which not only affects their stay in the neonatal intensive care unit (NICU), but also potentially their long-term health [6,7].

Since neurodevelopmental outcomes have been linked to postnatal growth, a single institutional quality improvement initiative, in a tertiary NICU, was designed to identify potential factors affecting postnatal growth rates and, thereby, to identify opportunities for positive change. The factors investigated were focused around early nutritional practices, including days of parenteral nutrition, feeding type: mother’s own milk or donor milk, and fortification: highest calories achieved at different time points in the first postnatal month. At the conception of this project, we parameterized postnatal growth as the change in weight *z*-scores from birth to discharge among preterm infants who were discharged by 40 weeks’ gestational age. In particular, we aimed to investigate whether feeding practices in the first postnatal month are associated with growth in this population of premature infants. In our investigation of the factors associated with high or low change in weight *z*-score, we encountered two interesting phenomena with respect to the chosen outcome of interest and its association with in-hospital feeding practices. In this paper, we aim to evaluate the role of selection bias in studies of preterm infant growth trajectory and the potential for confounding due to cohort effect.

## 2. Materials and Methods

### 2.1. Study Population

The subjects in this retrospective observational study were infants born between 1 June 2016 and 31 May 2018 and admitted into the NICU at Yale New Haven Hospital. Our two inclusion criteria were (1) gestational age at birth between 22 and 34 weeks, and (2) hospital discharge before or at 40 weeks’ gestational age. Birth weight was intentionally not used in assessment of eligibility to avoid the skew towards infants born smaller at 30 to 34 weeks’ gestational age that occurs when very low birth weight (VLBW) is an inclusion criterion.

### 2.2. Outcome and Predictors of Interest

As this study was part of a quality improvement initiative to improve in-hospital growth of preterm infants, we chose weight *z*-score, calculated by the Fenton growth chart as the primary measure of growth [8]. We used weight *z*-score at birth and discharge, obtained by inputting infant’s sex, gestational age at birth and discharge, and respective infant weights. We then calculated postnatal in-hospital growth as the difference between discharge and birth weight *z*-scores. Infants were grouped into quartiles of the observed change in *z*-scores. Following the example set by Ehrenkranz et al. of comparing growth trajectory by quartiles, we used the first and last quartiles of this change in weight *z*-score to define two groups: low growth quartile (LGQ) and high growth quartile (HGQ) [1]. We focused on the two extremes because comparing characteristics of preterm infants with the lowest and highest growth would identify factors associated with these patterns of postnatal growth in this population of infants, and thus help identify potential opportunities for clinical practice improvement.

To identify correlates of high and low growth, we compared the two groups with respect to infant demographic and clinical characteristics, as well as feeding experiences among these infants. Comorbidities commonly associated with growth failure such as intraventricular hemorrhage (IVH), necrotizing enterocolitis (NEC), patent ductus arteriosus (PDA), and small-for-gestational age (SGA) status at birth were collected, as well as head circumference and length at birth and discharge. The comorbidities were collected by Internal Classification of Diseases (ICD) codes for the respective diagnosis. For feeding experiences, we collected first postnatal day without parenteral nutrition (PN), as a proxy for days to full enteral feeds. Additionally, we collected the highest concentration of enteral calories received (<24 kcal, 24 kcal, or >24 kcal) and types of feeds the infant was receiving, i.e., mother’s own milk (yes/no) or donor milk (yes/no) per week over the first postnatal month. The feeds were not quantified by amount. They were counted as “yes” if any mother’s own milk or donor milk were received by the infant. Calories were based on documentation from the medical record system, as less than 24 kcal, 24 kcal, or greater than 24 kcal. Less than 24 kcal included both 22 kcal or 20 kcal (no fortification) feeds, and greater than 24 kcal included feeds with any fortification over 24 kcal. Standard fortification is used in the unit where this study was performed, as opposed to adjusted or targeted with recommendation for addition of human milk fortification at 100 mL/kg/day enteral feed volume. There was no data collected regarding the specific calorie content in each category or the type of fortification used.

In addition to the comparison of change in weight *z*-score, weight gain was compared as growth velocity. Average growth velocity was calculated as g/kg/day from birth to hospital discharge using the exponential model [9]. Calculation and comparison of growth velocity allowed for comparison of extrauterine growth in a manner not related to the reference growth curve. The referent population of sex-specific infant weights at specific birth gestational ages that form intrauterine reference growth curves, such as the Fenton growth chart, are developed from serial cross-sectional data collected at birth. This means that a weight *z*-score for an infant of a given sex and gestational age will come from the underlying distribution of infants of that sex who are born at that gestational age, i.e., an infant’s trajectory of change in his or her *z*-score up to the term gestational age is assumed to be composed of serial *z*-scores reflecting how well that infant is doing relative to a fetus born at that gestational age. However, an infant’s gestational age at birth can be viewed as exhibiting a cross-sectional, or cohort, effect on his or her longitudinal trajectory of change in growth. Therefore, simply using weight *z*-scores obtained from serial cross-sectional data (cohort-averaged growth) has the potential to conflate the cross-sectional or cohort effect of gestational age at birth and the longitudinal effect of gestational age, expressed as the postnatal day from birth. Therefore, our second growth outcome was the average change in infant’s weight across gestational age, separating the cohort-averaged effect of gestational age on weight into cross-sectional and longitudinal effects. Similar to the primary outcome of change in weight *z*-score from baseline to discharge, we investigated whether patient demographic and clinical characteristics, as well as feeding practices were associated with the mean change in weight. All data was collected via electronic medical records using the Joint Data Analytics Team at our institution.

### 2.3. Statistical Analysis

Descriptive summary statistics, such as mean (standard deviation), median (range), as well as count (percent) were used to summarize characteristics of preterm infants. Distributions of continuous variables were visually examined for deviations from normality and for presence of potential outliers using box plots and Q-Q plots. We tabulated demographic and clinical characteristics, as well as feeding practices by the quartiles of change in weight *z*-score and used parametric (Student’s *t*-test) and non-parametric (Mann–Whitney U test) approaches to compare continuous variables between LGQ and HGQ groups. Chi-square or Fisher’s exact tests (as appropriate for expected counts of less than five) were used to compare the two extreme groups of postnatal growth for categorical variables. We visually summarized the dependency of the change in weight *z*-score from baseline to discharge on gestational age at birth and birth weight *z*-score, using a three-dimensional figure resembling a heat-map.

Because infants were not randomized to receive mother’s own milk in the first postnatal month, other factors associated with both—the probability of receiving mother’s own milk (the exposure variable of interest) and change in weight *z*-score (the primary outcome)—were known to confound the association between type of feeding and change in weight *z*-score. This is described as selection bias. To balance groups of infants receiving and not receiving mother’s own milk in the first postnatal month with respect to confounders, we used augmented inverse probability weighting (AIPW), to estimate the average causal or treatment effect (ATE) of mother’s own milk on the change in weight *z*-score in the full cohort [10,11,12,13]. We first estimated each infant’s propensity score, which is the conditional probability of receiving mother’s own milk by postnatal day 28, using multiple logistic regression with race, birthweight *z*-score, gestational age at birth, growth velocity in kg/g/d, and days on PN. Inverse probability weights were then calculated by taking the inverse of the propensity scores and were applied in modeling the causal association between the mother’s own milk variable and change in weight *z*-score using linear regression. An absolute value of a standardized difference of <0.10 was used to indicate balance in potential confounders [14]. We used 1000 bootstrap samples to obtain standard errors and confidence intervals via the bias-corrected percentile method (e.g., taking 2.5th and 97.5th percentiles of the bootstrapped distribution of ATE) to get 95% confidence interval (95% CI).

Mean change in weight across gestational age (up to the second-degree polynomial effect) was modeled using linear mixed effects (LME) approach, with race, sex, mother’s own milk by postnatal day 28, and highest number of calories achieved per week in the first month (reference: >24 kcal/oz), with a random intercept and a random slope for each infant to account for the within-infant correlation in weights across gestational age. Likelihood ratio test (LRT with restricted maximum likelihood, REML) using a mixture of two chi-square distributions for the level of significance confirmed that we needed to retain both random effects. To examine whether a model with the decomposed effect of gestational age (cross-sectional and longitudinal) fits the data better than a model with time-varying gestational age (cohort-averaged), we tested the equality of the cross-sectional and longitudinal parameters via LRT (with full maximum likelihood, ML). To show all three possible effects of gestational age on change in weight, we plotted: (1) smoothed mean trajectories of change in weight over gestational age by birth gestational age, (2) the cross-sectional effect of gestational age by smoothing over weights at birth gestational age only, and (3) the cohort-averaged effect by smoothing over weight values across all gestational ages. Smoothed trajectories were obtained using locally estimated scatterplot smoothing (LOESS), with second-degree polynomial effect of gestational age at the center of neighboring observations and the smoothing parameter set to 0.6.

Statistical significance was established at the two-sided alpha of 0.05, but because such a threshold is a convention and we did not want to discount a clinically meaningful effect, we also reported 95% CIs and noted *p* < 0.15 because we did not power our study. Analyses were performed using SAS 9.4 (Cary, NC, USA) and R-statistical software.

## 3. Results

**Figure 1:**
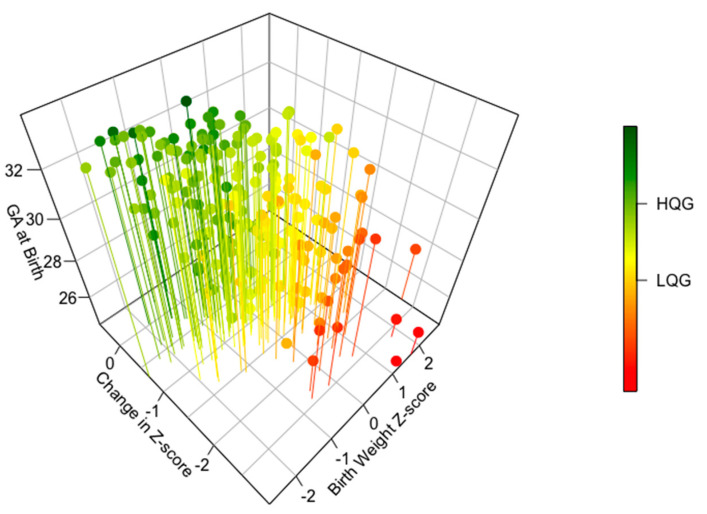
Associations of gestational age and birth weight *z*-score with change in weight *z*-score from birth to discharge (Number = 204).

Our initial search yielded 204 preterm infants, with a mean birth gestational age of 30.3 weeks (SD = 2.24), and a mean birth weight of 1393.9 g (SD = 379.5). No outlying values were identified. For the cohort, the mean and the median change in weight *z*-score was −1.0 (SD = 0.6, range −3.0, 0.5). As described, the cohort was divided into quartiles based on change in weight *z*-score. A change in weight *z*-score of ≤−1.34 defined the LGQ. A change in weight *z*-score of >−0.57 defined the HGQ. The complex dependency of the change in weight *z*-score from birth to discharge on the birth gestational age and birth weight *z*-score is illustrated in Figure 1. In particular, infants born earlier in the gestational period tended to have higher birth weight *z*-scores and less favorable change in weight *z*-scores as shown by more negative values (pins on the red color spectrum). In contrast, infants born later had lower weight *z*-scores at birth and more favorable change in *z*-score at discharge (pins on the green color spectrum).

When comparing characteristics of HGQ and LGQ groups, no statistically significant differences (*p* > 0.05) were found with respect to sex or race, as well as comorbidities in this cohort of preterm infants with hospital discharge prior to or at 40 weeks’ gestational age (Table 1). As shown in Table 2, there were notable between-group differences at birth and discharge. In particular, infants in the HGQ group compared to the LGQ group had significantly higher gestational age at birth, shorter length of stay (LOS), and lower gestational age at discharge. As shown in Table 3, though the birth and discharge gestational ages were significantly different, birth and discharge weights were not. In comparison of other growth parameters, all were similar, except birth head circumference was significantly higher in the HGQ compared to the LGQ group. Similar to the change in weight *z*-score by which these two groups were defined, both change in length *z*-score and change in head circumference *z*-score showed less of a loss through the hospitalization in the HGQ compared to the LGQ group. Growth velocity calculation, which was used as a method to avoid the cross-sectional component of the *z*-score calculation which relies on fetal growth, was significantly higher in the HGQ cohort compared to the LGQ cohort. Data from all quartiles is shown in Table A1, Table A2 and Table A3 in Appendix A.

The nutritional practices of earlier enteral nutrition, less mother’s own and donor milk feeding, and higher enteral caloric density were all significantly associated with higher growth when expressed as the change in weight *z*-score. This was demonstrated by HGQ infants, as compared to LGQ, having significantly fewer days to full enteral nutrition (median of 7 days vs. 10 days, *p* < 0.01), and higher enteral caloric density in the first postnatal week (≥24 cal/oz 62.7% vs. 23.5%, *p* < 0.001), lower mother’s own milk intake in the first postnatal week (82.4% vs. 98.0%, *p* = 0.01) and month (76.1% vs. 95.8%, *p* = 0.01), and lower donor milk intake in the first week (23.5% vs. 52.9%, *p* < 0.01) and month (2.2% vs. 14.7%, *p* = 0.06). After accounting for confounders, the average change in weight *z*-score from birth to discharge was −1.0 vs. −1.2 if receiving mother’s own milk by postnatal day 28 vs. not, respectively. This translated into ATE = 0.2 (95%CI −0.05, 0.31; *p* = 0.14), meaning that receiving mothers’ own milk may translate into higher growth change. Additionally, in this model, for every one week of increase in gestational age at birth, the change in *z*-score favorably changed by an average of 0.16 (*p* < 0.001).

**Figure 2:**
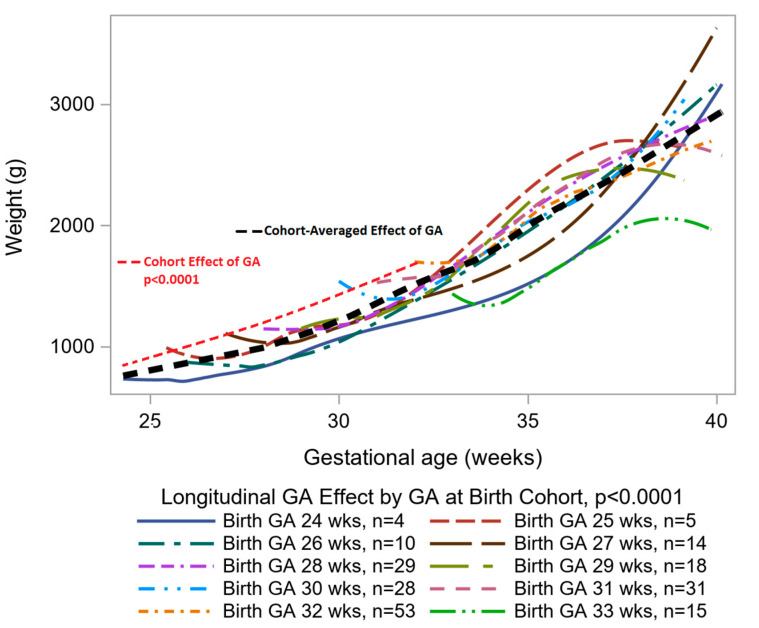
Decomposition of cohort-averaged effect of gestational age on change in weight. *p*-values obtained from linear mixed effects modeling (LME).

**Figure 3:**
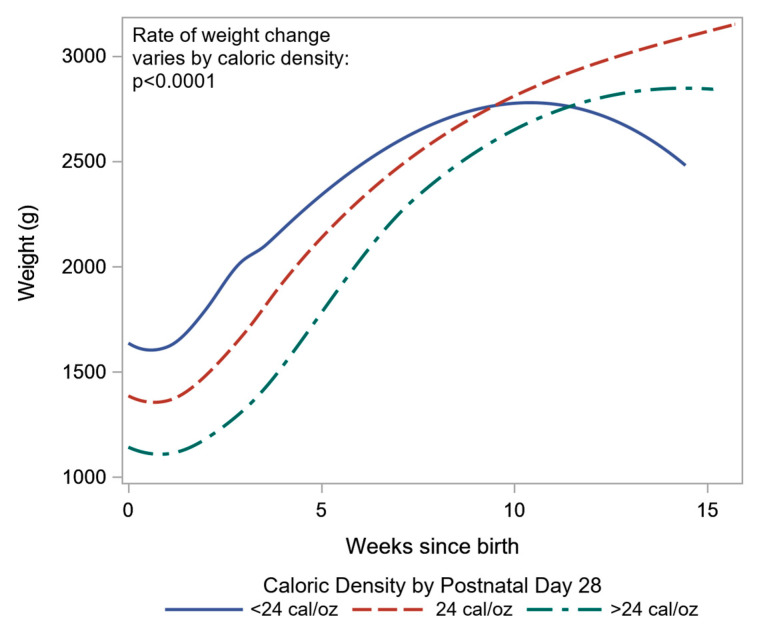
Smoothed trajectories of change in weight during postnatal period in neonatal intensive care unit (NICU) by caloric density on day 28 of life. *p*-value obtained from the interaction effect of weeks since birth and caloric density in LME.

Our analysis of weight change across gestational age revealed that gestational age at birth played an important role in the subsequent weight change during postnatal stay in the hospital (*p* < 0.0001). Therefore, we need both, the cross-sectional and the longitudinal effects of gestational age, when modeling growth as expressed by infant’s weight. Figure 2 shows the cohort-averaged effect of gestational age on weight, as is assumed in the *z*-score calculations, can be separated into a higher rate of change in weight as the gestational age at birth increases and into a slower rate of change based on the longitudinal or time since birth effect of time. Therefore, when we use the change in *z*-score to track growth, we effectively expect weight to go up at a higher rate than is explained by the longitudinal decomposition of change in weight, especially in the first two postnatal weeks, and, hence, the resulting change in *z*-scores is more negative than it should be. Of note, the only other significant predictor of weight change across gestational age in the LME models was the caloric density by postnatal day 28 (Figure 3). Infants receiving <24 cal/oz had higher birth weight (Figure 3) than infants on higher caloric density, but the difference in weight narrowed across time, especially between weeks 5 and 10 during the postnatal period (*p* < 0.0001).

## 4. Discussion

This study was initially designed to investigate feeding practices in the first postnatal month that may be associated with improved growth, in an attempt to find specific areas to target for quality improvement practices. Past studies have demonstrated a positive association between preterm infant in-hospital growth trajectory and neurodevelopment [1,2,3,4,5], and although these outcomes are out of the scope of this study, these previous studies reveal the importance of neonatal nutrition, not only for growth in the NICU, but potentially beyond this short-term goal.

From our results, we found shorter duration to full enteral feeds and likewise increased enteral calories, in other words, early fortification, in the first postnatal week in the HGQ group. These results are not surprising as they are similar to Culpepper et al. who found a 15% improvement in 28-day weight gain with implementation of a feeding guideline to start feeding and to achieve full feeding sooner in a cohort of VLBW infants [15]. In addition, the HGQ had a significantly lower proportion of infants on mother’s own milk and donor human milk. Though the proportion of infants receiving formula was not collected, since the infants in the HGQ were more likely to receive enteral nutrition, and it was less likely to be mother’s own milk or donor human milk, it is assumed to be enteral intake of infant formula. This result, also, is not unexpected as preterm infants are known to demonstrate higher weight gain with infant formula compared to human milk [16,17]. Of note, when accounting for confounders, infants receiving mother’s own milk by postnatal day 28 demonstrated a more favorable change in weight *z*-score compared to infants who did not receive mother’s own milk. However, a larger study is required to further investigate the association between mother’s own milk intake and preterm infant growth.

Although our results indicated that better growth trajectory, as measured by the change in weight *z*-score, was associated with shorter time to full enteral nutrition, and higher enteral energy density, or early fortification, these associations are affected by selection bias. Interestingly, in this study, subject selection intentionally included infants based on birth gestational age rather than on birth weight to avoid the skew that can occur at the higher gestational ages in VLBW infant studies without attention to gestational age. This study included infants born 22 to 34 weeks’ gestational age to provide a comprehensive assessment of growth in this population instead of only including the VLBW infants in the 32–34 weeks’ gestational age range as commonly occurs in VLBW infant studies. Instead of correcting the inherent bias of VLBW infant-focused research, our study introduced new selection bias due to the great differences in receipt of nutritional care as well as great differences in growth potential in infants from 22 to 34 weeks’ gestational age as shown in Figure 1. Of note, this selection bias may not have been evident if our study had only included birth weight and discharge weight as these variables are not significantly different between the HGQ and LGQ groups. It was attention to birth weight *z*-score and gestational age that made the selection bias evident.

In our study, the comparison of high and low change in *z*-score in preterm infants was strongly related to birth gestational age and birth weight despite the postnatal exposures, and, therefore, selection bias had a great effect on the results. For example, for every 1 week increase in gestational age at birth, the change in *z*-score changed by an average of 0.16. Therefore, as our analysis of decomposition of the cohort-averaged effect of gestational age on weight suggests, when we track growth as the difference in successive *z*-scores, the resulting change is more negative than it should be because individual *z*-scores were obtained from weights of infants from different cohorts defined by gestational age at birth.

This study identifies the innate difficulties with investigation of optimal growth trajectory both in research and in clinical care. Though studies demonstrate a positive association between preterm infant in-hospital growth trajectory and neurodevelopment [1,2,3,4,5], how to translate this research data into a clinical recommendation for growth goals remains difficult. Common methods of preterm infant growth monitoring include serial measurements plotted on intrauterine growth curves (Fenton, Olsen) [8,18] or preterm infant growth curves (Intergrowth) [19] or calculation of weight gain velocity [9,20]. Monitoring of growth on growth curves can be at a specific gestational age or by calculation of longitudinal changes [21]. Intrauterine growth curves illustrate the gold standard of preterm infant growth which is to match fetal growth. However, an innate weakness of the intrauterine growth curve is that it demonstrates fetal growth by measuring newborn preterm infants. Therefore, two critical weaknesses are (1) the data is serial cross-sectional instead of longitudinal and (2) the data represents infants who are no longer in utero—thereby, an insult to fetal health occurred.

Despite these inherent weaknesses with referencing intrauterine growth and the known importance of growth for preterm infant outcomes, intrauterine growth curves remain the standard in neonatal care. In clinical care, infant growth parameters are commonly plotted on percentile lines which provide a visual of the growth trajectory. Conversely, Rochow et al. has described how this visual does not promote reliable interpretation of growth trajectory [22,23]. Instead, *z*-scores, calculated from the same reference growth curves, provide a reliable measure for use in mathematical equations, such as change in *z*-score over time. However, despite the mathematical reliability of *z*-score difference over time, the inherent error in measuring longitudinal data on a cross-sectional reference persists. Preterm infant growth curves based on longitudinal growth, such as the Intergrowth curve, address this problem [19]. However, with fetal growth as the established gold standard, preterm infant growth curves must be related to preterm infant outcomes before they can be reliably used in clinical care. Furthermore, there are inconsistencies among what defines healthy growth not only with how to best measure, but with where to start tracking the measurements and whether postnatal diuresis or weight loss is inevitable or not [22,24,25], which require further investigation.

The use of growth velocity calculations provides a method to compare growth while avoiding the weaknesses of growth curves. However, with the common post-birth weight loss, identifying where to initiate this calculation, i.e., at 2 postnatal weeks or once the infant has returned to birth weight, is complicated. Various mathematical equations for calculation are available including an exponential model validated by Patel et al. [9]. In comparison of three growth velocity calculations, Fenton et al. found the exponential model and the average (two-point) calculation to show agreement [20]. As to how growth velocity may be used to identify appropriate preterm growth, Ehrenkranz et al. demonstrated improved neurodevelopmental outcomes at 18–22 months’ corrected age with weight gain of an average of 21.2 g/kg/day from when the infant regained birth weight until discharge, transfer, 120 days, or until a weight of 2 kg was reached [1]. However, this long duration of measurement is difficult to translate to shorter durations of growth velocity measurement as needed for clinical care decisions during hospitalization. Other innovative measures such as weight gain ratios [23] or growth velocity calculations with postnatal adjustment are relatively new methods for preterm infant growth monitoring and, therefore, require further investigation as methods to control for the errors in preterm infant growth assessment [23,26]. In our study, the HGQ demonstrated significantly higher growth velocity when compared to the LGQ group.

Some limitations with this study include the innate limitations with retrospective data collection, the biases as described above, lack of detailed information on feeding practices of different gestational ages at the time the data was collected, and the absence of nutritional laboratory data. Although we saw improved change in weight *z*-score with increased calories in the first postnatal week, the type of calories added were not specified either (i.e., human milk fortifier, formula powder added to human milk, or calorically-dense preterm formula). In addition, there was not adequate sample size to stratify the data by gestational age at birth. An infant born at 22 weeks’ gestational age is vastly different from an infant born at 32 weeks’ gestational age, including enteral nutrition exposures and, likely, as shown in our study, in growth trajectory potential. For comorbidities, reliance on ICD codes may have missed non-coded diagnoses, and data was not collected on other common comorbidities such as chronic lung disease or metabolic bone disease. In addition, all of the data was from a single center.

## 5. Conclusions

The results of this study demonstrate a significant role of early factors in hospital growth trajectory. When evaluating these preterm infants, infants with higher gestational age had lower *z*-scores at birth and demonstrated better growth trajectories, defined by change in weight *z*-score. These patterns observed in a preterm infant cohort should be taken into consideration when evaluating the association between preterm infant growth and infant outcomes. Due to selection bias as seen in our study population, it is difficult to interpret these results. In addition, the cohort effect due to varying gestational age at birth, as seen in this method of tracking growth, also must be taken into consideration when investigating studies of relating growth and outcomes.

While patterns of weight *z*-scores over time are useful guides for physical growth progression and monitoring nutritional practices in this population, especially early after birth, the inherent bias and cohort effect may have a significant impact on the growth outcomes. Therefore, the best way to track this growth and to investigate optimal nutritional interventions remain critical research questions.

## Figures and Tables

**Table 1:** Characteristics compared between lowest growth quartile (LGQ) and highest growth quartile (HGQ).

Characteristic	LGQ (Number = 51)	HGQ (Number = 51)	*p*-Value ^5^
Sex (female/male)	25/26	21/30	0.43
Race (number)			0.86
Black or African American	10	12	
White or Caucasian	24	24	
Other	17	15	
Comorbidities (yes/no)			
Grade III IVH ^1^	1/50	0/51	1.00
Grade IV IVH	1/50	1/50	1.00
NEC ^2^	0/51	0/51	1.00
PDA ^3^	5/46	3/48	0.72
SGA ^4^	1/50	3/48	0.62

^1^ IVH = intraventricular hemorrhage, ^2^ necrotizing enterocolitis, ^3^ patent ductus arteriosus, ^4^ small for gestational age, and ^5^ *p*-value calculated by chi-square and Fisher’s exact tests. Comorbidity information was not available on all subjects.

**Table 2:** Gestational age at birth and discharge, and length of stay compared between LGQ and HGQ.

	LGQ (Number = 51)	HGQ (Number = 51)	*p*-Value ^1^
Gestational Age at Birth (weeks)			
Median (Range)	29.4 (24.3–32.9)	32.1 (26.0–33.9)	<0.001
Gestational Age at Discharge (weeks)			
Median (Range)	37.6 (35.0–39.3)	36.6 (35.4–40.0)	0.021
Length of Stay (days)			
Median (Range)	59.0 (15.0–100.0)	32.0 (18.0–93.0)	<0.001

^1^*p*-value by Mann–Whitney U test.

**Table 3:** Growth parameters (weight, length, and head circumference) compared between LGQ and HGQ.

Growth Parameter	LGQ (Number = 51)	HGQ (Number = 51)	*p*-Value ^1^
Birth Weight (g)			
Mean (SD) ^2^	1371.86 (389.62)	1428.04 (357.61)	0.45
Birth Weight *z*-score			
Mean (SD)	0.53 (0.89)	−0.67 (0.80)	<0.001
Birth Length (cm)			
Mean (SD)	38.76 (3.79)	39.98 (3.38)	0.09
Birth Length *z*-score			
Mean (SD)	0.46 (1.01)	−0.39 (0.88)	<0.001
Birth Head Circumference (cm)			
Mean (SD)	26.91 (2.23)	27.88 (2.18)	0.028
Birth Head Circumference *z*-score			
Mean (SD)	0.35 (0.87)	−0.52 (1.06)	<0.001
Discharge Weight (g)			
Mean (SD)	2451.12 (372.19)	2431.57 (444.86)	0.81
Discharge Weight *z*-score			
Mean (SD)	−1.26 (0.88)	−0.96 (0.87)	0.09
Discharge Length (cm)			
Mean (SD)	46.25 (2.55)	45.30 (2.37)	0.06
Discharge Length *z*-score			
Mean (SD)	−0.90 (1.13)	−0.98 (0.94)	0.70
Discharge Head Circumference (cm)			
Mean (SD)	31.98 (1.74)	32.00 (1.49)	0.94
Discharge Head Circumference *z*-score			
Mean (SD)	−0.99 (0.97)	−0.66 (0.82)	0.07
Growth Velocity (gm/kg/day)			
Median (Range)	10.7 (0.9–13.2)	14.2 (10.2–20.8)	<0.001
Change in Weight *z*-score			
Mean (SD)	−1.79 (0.41)	−0.29 (0.24)	<0.001
Change in Length *z*-score			
Mean (SD)	−1.36 (0.94)	−0.59 (0.65)	<0.001
Change in Head Circumference *z*-score			
Mean (SD)	−1.35 (0.85)	−0.14 (0.70)	<0.001

^1^*p*-value calculated by Student’s *t*-test. ^2^ standard deviation.

## Appendix A

Table A1, Table A2 and Table A3 in Appendix A correlate with Table 1, Table 2 and Table 3 in this manuscript, representing the same characteristics and comparisons, but consisting of data from all four quartiles.

**Table A1:** Characteristics of preterm infants by quartile of growth trajectory.

Characteristic	Z_diff_ ≤ −1.34 ^1^ (Number = 51)	−1.34 < Z_diff_ ≤ −0.93 ^1^ (Number = 51)	−0.93 < Z_diff_ ≤ −0.57 ^1^ (Number = 51)	Z_diff_ > −0.57 ^1^ (Number = 51)	Total (Number = 204)	*p*-Value ^6^
Sex (female/male)	25/26	22/29	28/23	21/30	96/108	0.5
Race (number)						0.53
Black or African American	10	12	16	12	50	
White or Caucasian	24	25	27	24	100	
Other	17	14	8	15	54	
Comorbidities (yes/no)						
Grade III IVH ^2^	1/50	1/50	0/51	0/51	2/202	1.00
Grade IV IVH	1/50	0/51	0/51	1/50	2/202	1.00
NEC ^3^	0/51	1/50	0/51	0/51	1/203	1.00
PDA ^4^	5/46	4/47	6/45	3/48	18/186	0.84
SGA ^5^	1/50	2/49	6/45	3/48	12/192	0.24

^1^ Change in weight *z*-score: Z_diff_ = Z_discharge_ − Z_birth_, ^2^ IVH = intraventricular hemorrhage, ^3^ necrotizing enterocolitis, ^4^ patent ductus arteriosus, ^5^ small for gestational age, and ^6^ *p*-value calculated by chi-square and Fisher’s exact tests. Comorbidity information was not available on all subjects.

**Table A2:** Preterm infants’ gestational age at birth and discharge, and length of stay compared among quartiles of growth trajectory.

	Z_diff_ ≤ −1.34 ^1^ (Number = 51)	−1.34 < Z_diff_ ≤ −0.93 ^1^ (Number = 51)	−0.93 < Z_diff_ ≤ −0.57 ^1^ (Number = 51)	Z_diff_ > −0.57 ^1^ (Number = 51)	Total (Number = 204)	*p*-Value ^2^
Gestational Age at Birth (weeks)						
Median (Range)	29.4 (24.3–32.9)	30.1 (24.9–33.6)	31.4 (24.4–33.9)	32.1 (26.0–33.9)	30.9 (24.3–33.9)	<0.001
Gestational Age at Discharge (weeks)						
Median (Range)	37.6 (35.0–39.3)	36.9 (35.0–39.9)	36.4 (35.0–40.0)	36.6 (35.4–40.0)	36.7 (35.0–40.0)	0.11
Length of Stay (days)						
Median (Range)	59.0 (15.0–100.0)	46.0 (17.0–105.0)	39.0 (16.0–109.0)	32.0 (18.0–93.0)	44.0 (15.0–109.0)	<0.001

^1^ Change in weight *z*-score: Z_diff_ = Z_discharge_ – Z_birth_, ^2^ *p*-value by Kruskal–Wallis test.

**Table A3:** Growth parameters (weight, length, and head circumference) compared among quartiles of growth trajectory.

Growth Parameter	Z_diff_ ≤ −1.34 ^1^ (Number = 51)	−1.34 < Z_diff_ ≤ −0.93 ^1^ (Number = 51)	−0.93 < Z_diff_ ≤ −0.57 ^1^ (Number = 51)	Z_diff_ > −0.57 ^1^ (Number = 51)	Total (Number = 204)	*p*-Value ^2^
Birth Weight (g)						
Mean (SD) ^3^	1371.86 (389.62)	1350.90 (345.53)	1424.94 (425.38)	1428.04 (357.61)	1393.94 (379.46)	0.67
Birth Weight *z*-score						
Mean (SD)	0.53 (0.89)	−0.08 (0.77)	−0.28 (1.02)	−0.67 (0.80)	−0.12 (0.97)	<0.001
Birth Length (cm)						
Mean (SD)	38.76 (3.79)	38.59 (3.62)	39.45 (4.09)	39.98 (3.38)	39.20 (3.75)	0.21
Birth Length *z*-score						
Mean (SD)	0.46 (1.01)	−0.11 (1.03)	−0.18 (1.29)	−0.39 (0.88)	−0.06 (1.10)	<0.001
Birth Head Circumference (cm)						
Mean (SD)	26.91 (2.23)	27.11 (2.09)	27.75 (2.53)	27.88 (2.18)	27.41 (2.28)	0.08
Birth Head Circumference *z*-score						
Mean (SD)	0.35 (0.87)	−0.09 (0.97)	−0.14 (1.19)	−0.52 (1.06)	−0.10 (1.07)	<0.001
Discharge Weight (g)						
Mean (SD)	2451.12 (372.19)	2397.10 (376.48)	2410.67 (458.92)	2431.57 (444.86)	2422.61 (412.40)	0.92
Discharge Weight *z*-score						
Mean (SD)	−1.26 (0.88)	−1.22 (0.75)	−1.06 (0.98)	−0.96 (0.87)	−1.13 (0.88)	0.28
Discharge Length (cm)						
Mean (SD)	46.25 (2.55)	45.50 (2.45)	45.50 (2.75)	45.30 (2.37)	45.64 (2.54)	0.25
Discharge Length *z*-score						
Mean (SD)	−0.90 (1.13)	−1.05 (0.96)	−0.92 (1.11)	−0.98 (0.94)	−0.96 (1.03)	0.88
Discharge Head Circumference (cm)						
Mean (SD)	31.98 (1.74)	31.92 (1.44)	31.98 (1.77)	32.00 (1.49)	31.97 (1.60)	0.99
Discharge Head Circumference *z*-score						
Mean (SD)	−0.99 (0.97)	−0.88 (0.81)	−0.71 (1.03)	−0.66 (0.82)	−0.81 (0.92)	0.25
Growth Velocity (gm/kg/day)						
Median (Range)	10.7 (0.9–13.2)	11.7 (4.2–16.6)	12.7 (7.5–16.7)	14.2 (10.2−20.8)	12.1 (0.9 – 20.8)	<0.001
Change in Length *z*-score						
Mean (SD)	−1.36 (0.94)	−0.94 (0.85)	−0.74 (0.76)	−0.59 (0.65)	−0.91 (0.85)	<0.001
Change in Weight *z*-score						
Mean (SD)	−1.79 (0.41)	−1.14 (0.11)	−0.79 (0.10)	−0.29 (0.24)	−1.0 (0.60)	<0.001
Change in Head Circumference *z*-score						
Mean (SD)	−1.35 (0.85)	−0.78 (0.73)	−0.57 (0.77)	−0.14 (0.70)	−0.71 (0.87)	<0.001

^1^ Change in weight *z*-score: Z_diff_ = Z_discharge_ − Z_birth_; ^2^ *p*-Value calculated by Analysis of Variance (ANOVA) test. ^3^ standard deviation.
